# Commencement Exercises of the Medical Department of the University of Buffalo

**Published:** 1855-03

**Authors:** 


*Commencement Exercises of the Medical Department of the University of Buffalo.*—This occasion was one of considerable interest. Townsend Hall was filled to overflowing with an intelligent audience. The degree of Doctor of Medicine was conferred on the following individuals:

Milan Baker, Rochester, N. Y.
Jeremiah Nathaniel Brown, Buffalo, N. Y.
Orville R. Flowers, Fowler, St. Lawrence Co., N. Y.
Alexander Grant, York, Livingston Co., N. Y.
William Howell, Buffalo, N. Y.
James T. Hubbard, Lockport, Niagara Co., N. Y.
James McLaren, Canada West.
Thomas Morrison, " "
Francis M. Perine, Dansville, Livingston Co., N. Y.
Eleazar Price, Buffalo, N. Y.
George Rais du Forburg, Buffalo, N. Y.
R. Stephenson, Adrian, Michigan.
A. Taylor, Cuba, Allegany Co., N. Y.
Webster Wynn, Lima, Livingston Co., N. Y.

The Honorary Degree of Doctor of Medicine was also conferred on Cornelius Faling, of Royalton, Niagara Co., N. Y.

The Thesis of Wm. Howell was ordered for publication by the Curators. The Thesis of George Rais du Forburg to receive honorable mention.

The services opened by an impressive prayer by the Rev. Dr. Thompson. The degrees were then conferred by the Hon. Millard Fillmore, Chancellor of the University. The Charge to the Graduates was delivered by the Prof. of Anatomy, and the benediction was pronounced by the Rev. Dr. Shelton. The music was under the direction of Mr. Poppenburg.

